# Housekeeping gene expression variability in differentiating and non-differentiating 3T3-L1 cells

**DOI:** 10.1080/21623945.2023.2235081

**Published:** 2023-07-20

**Authors:** Danang Dwi Cahyadi, Tomoko Warita, Nanami Irie, Kana Mizoguchi, Jiro Tashiro, Yoshinao Z. Hosaka, Katsuhiko Warita

**Affiliations:** a Joint Graduate School of Veterinary Sciences, Tottori University, Tottori, Tottori, Japan; b Division of Anatomy Histology and Embryology, School of Veterinary Medicine and Biomedical Sciences, IPB University, Bogor, West Java, Indonesia; c Department of Biomedical Sciences, School of Biological and Environmental Sciences, Kwansei Gakuin University, Sanda, Hyogo, Japan; d Graduate School of Science and Technology, Kwansei Gakuin University, Sanda, Hyogo, Japan; e Department of Bioresource Sciences, Faculty of Agriculture, Kyushu University, Fukuoka, Fukuoka, Japan; f Joint Department of Veterinary Medicine, Faculty of Agriculture, Tottori University, Tottori, Tottori, Japan

**Keywords:** adipocytes, differentiation, gene expression, normalization, reference gene

## Abstract

Normalization is a crucial step in gene expression analysis to avoid misinterpretation. Reverse transcription-quantitative polymerase chain reaction was used to measure the expression of 10 candidate housekeeping genes in non-differentiated (ND) and differentiated (DI) 3T3-L1 cells on days 5 and 10. We used geNorm, NormFinder, BestKeeper, RefFinder, and the ∆Ct method to evaluate expression stability. The findings revealed that (1) the expression levels of the reference genes changed over time, even in non-differentiating cells, and (2) peptidylprolyl isomerase A (*Ppia*) and TATA box-binding protein (*Tbp*) were stable reference genes for 10 days in both undifferentiated and differentiated 3T3-L1 cells. Notably, the expression of known reference genes in non-differentiating cells was altered throughout the experiment.

## Introduction

Obesity has become a global concern owing to its increasing prevalence [1] and its potential to increase the risk of metabolic diseases [2–4]. Consequently, the number of studies on obesity has increased worldwide [5,6]. Numerous studies have been conducted to understand the underlying mechanisms of adipocyte development and discover potential therapies for obesity. Various mouse-derived cell lines such as 3T3-L1 and its sublines 3T3-F442A [7,8], Ob17 [9], C3H10T1/2 [10], AP-18 [11], OP9 [12], and mouse embryonic fibroblasts (MEFs) [13] have been established as alternatives to primary cultures and animal models to study adipogenesis. Among these cell lines, 3T3-L1 is the most widely used for adipogenesis studies [14]. Adipogenesis, the process of adipocyte differentiation, is regulated at the molecular level by several key transcription factors, including peroxisome proliferator-activated receptor gamma (PPARγ) and CCAAT/enhancer binding protein alpha (CEBPα) [15]. Because multiple molecular events occur during adipogenesis, it is essential to evaluate the expression of various genes that underlie this process, which may be affected by adipogenesis.

Reverse transcription-quantitative polymerase chain reaction (RT-qPCR) has been widely utilized for mRNA quantification in gene expression analysis because of its high sensitivity and accuracy, as well as its ability to perform real-time analysis [16,17]. However, some technical issues need to be considered, including RNA extraction, handling, and storage, reverse transcription, and amplification efficiency. Therefore, appropriate normalization of the data against a valid reference gene, which could control possible experimental errors throughout the process [18], should be considered to ensure the reliability of the qPCR data, as described in the Minimum Information for Publication of Quantitative Real-Time PCR Experiments (MIQE) guidelines [19].

Several genes such as beta-actin (*Actb*), glyceraldehyde-3-phosphate dehydrogenase (*Gapdh*), and 18S ribosomal RNA (*Rn18s*) are commonly used as reference genes; however, their use has decreased as the utilization of other potential reference genes has increased since the publication of the MIQE guidelines [20]. There is no universal reference gene because it cannot be guaranteed that such a gene will be suitable for every experimental condition [21]. Thus, validation of suitable reference genes is an important step in gene expression studies using RT-qPCR. Although some studies have reported stable reference genes for RT-qPCR analysis in various 3T3-L1 adipocyte differentiation studies [22–25], validation of reference genes suitable for 3T3-L1 adipocyte studies that directly compare differentiating and non-differentiating conditions has not yet been performed. The expression levels of reference genes in 3T3-L1 cells may be altered throughout the experimental timeline under these two conditions; thus, normalizing the expression levels of target genes using unvalidated reference genes will lead to the misinterpretation of gene expression data.

In this study, we evaluated the expression patterns of genes that have been used or recognized as relatively stable in various experimental designs for 3T3-L1 cell differentiation studies [22,23,25,26], including 18S ribosomal RNA (*Rn18s*), hydroxymethylbilane synthase (*Hmbs*), peptidylprolyl isomerase A (*Ppia*), beta 2 microglobulin (*B2m*), and TATA box-binding protein (*Tbp*). In addition, the gene expression stability of the transferrin receptor (*Tfrc*), non-POU domain-containing octamer-binding protein (*Nono*), ribosomal protein L13A (*Rpl13a*), and two commonly used reference genes, beta-actin (*Actb*) and glyceraldehyde-3-phosphate dehydrogenase (*Gapdh*), were evaluated. The focus of our study was to evaluate the stability of gene expression and to validate the most suitable reference genes for 3T3-L1 adipocyte studies under both differentiation and non-differentiation conditions.

## Materials and methods

### Cell culture and differentiation

3T3-L1 preadipocytes were seeded at 5 × 10^4^ cells/mL/well in 12-well plates, at the same passage number (5), for all triplicate samples. Cells were cultured in Dulbecco’s modified Eagle’s medium (DMEM, 4.5 g/L glucose; FUJIFILM Wako Pure Chemical Corporation, Osaka, Japan) supplemented with 10% foetal bovine serum (FBS; Biosera, Ringmer, UK) and 1% penicillin and streptomycin (FUJIFILM Wako Pure Chemical Corporation) at 37°C under 5% CO_2_. The cells were treated with or without differentiation induction media and divided into five time-based groups: (i) control, day 0; (ii) non-differentiated (ND), day 5; (iii) ND, day 10; (iv) differentiated (DI), day 5; and (v) DI, day 10. Two days post-confluence (day 0), differentiation induction was started for the DI groups by replacing the medium with a differentiation medium containing 10 µg/mL insulin, 2.5 µM dexamethasone, and 0.5 mM 3-isobutyl-1-methylxanthine (AdipoInducer Reagent, Takara Bio, Shiga, Japan). The medium was replaced every 2 days with a medium containing 10 µg/mL insulin. The cells were cultured according to the manufacturer’s instructions. In the ND group, the medium was replaced with DMEM containing 10% FBS. Cells were collected for RNA extraction on day 0 (control) and days 5 and 10 for both the ND and DI groups. Cell cultures were prepared at the aforementioned time points for Oil Red O (ORO) staining. All cell culture experiments were performed in triplicate.

### Oil Red O staining

The cells were processed for ORO staining to verify lipid droplet accumulation and adipocyte differentiation. The cultured cells were washed with phosphate-buffered saline (pH 7.4) and fixed in 10% neutral-buffered formalin (pH 7.4; FUJIFILM Wako Pure Chemical Corporation) at a temperature ranging from 22°C to 24°C overnight. ORO staining solution (Lipid Assay Kit; Cosmo Bio, Tokyo, Japan) was freshly prepared by mixing the solution with distilled water (dH_2_O) at a ratio of 3:2. The working solution was filtered and stained according to the manufacturer’s protocol. The cells were then washed thrice with dH_2_O and air-dried. ORO solution was added to the culture plates, and the cells were incubated at room temperature for 1 h on an orbital shaker. After the ORO solution was removed, the stained cells were washed three times with dH_2_O, and images of the cells were captured using an inverted light microscope (Olympus IX71; Olympus, Tokyo, Japan). A semi-quantitative method was employed to determine lipid droplet accumulation. Isopropanol was used to extract the dye from the stained lipid droplets and the absorbance of the dye was measured at 492 nm using a microplate reader (Sunrise Remote; Tecan Austria GmbH, Grödig, Austria).

### RT-qPCR

Total RNA was isolated from the cells using the ISOSPIN Cell & Tissue RNA kit (Nippon Gene, Tokyo, Japan), and 500 ng of RNA with an A260/280 ratio ranging from 2.08 to 2.13 was reverse transcribed for cDNA synthesis using ReverTra Ace® qPCR RT Master Mix with gDNA Remover (Toyobo, Osaka, Japan) according to the manufacturer’s instructions. FastStart Essential DNA Green Master reaction mix (Roche Diagnostics GmbH, Mannheim, Germany), with a final volume of 20 µL for each sample, was used for qPCR analysis, which was performed on a LightCycler® Nano Real-Time PCR Instrument (Roche Diagnostics GmbH). The following 10 reference genes were analysed: *Actb*, *Gapdh*, *Rn18s*, *Hmbs*, *Ppia*, *B2m*, *Tbp*, *Tfrc*, *Nono*, and *Rpl13a*. We ensured that the primer efficiencies were following those in the LightCycler® Real-Time PCR Systems-Application Manual, which stipulates that PCR efficiency should be at least 85%. The PCR efficiencies of all primer sets used in this study varied between 87.6% (*Gapdh*) and 102.2% (*Tfrc*), which were within the acceptable range [27]. Moreover, the standard curves showed strong linearity with *r*^2^ values, ranging from 0.9838 (*B2m*) to 0.9999 (*Gapdh*), which is within the acceptable values for RT-qPCR reactions (*r*^2^ ≥ 0.98) [28,29]. Detailed information, including sequences, accession numbers, product sizes, and efficiencies of the primer sets for the 10 reference genes used for RT-qPCR, are presented in Table 1. The following primers were used for leptin (*Lep*) expression analysis: forward, 5′-TCCCTGCCTCAGACCAGTG-3′ and reverse, 5′-TAGAGTGAGGCTTCCAGGACG-3′ [31]. Two adipogenic transcription factors, *Pparg* and *Cebpa*, were evaluated. The following primers were used for the target genes: *Pparg* forward, 5′-CAAGAATACCAAAGTGCGATCAA-3′ and reverse, 5′-GAGCAGGGTCTTTTCAGAATAATAAG-3′ [32]; *Cebpa* forward, 5′-CTAGGAGATTCCGGTGTGGC-3′ and reverse, 5′-CCCGAGAGGAAGCAGGAATC-3′ [33]. The expression levels of all genes provided in the present study were automatically determined using the LightCycler® Nano Real-Time PCR System (Roche Diagnostics) based on the calibration curve from each qPCR. As previously mentioned, it is important to normalize gene expression to account for possible technical factors. Therefore, the expression of each gene in the analysis was referred to as a non-normalized expression.

**Table 1.** Mouse cDNA primer sequences of the 10 reference genes used for RT-qPCR.

Gene symbol	Primer sequences (5’−3’)	Accession number	Product size (bp)	Primer efficiency (%)	*r*^2^ value	References of primer sequences
*Actb*	F: AGCCATGTACGTAGCCATCC	NM_007393.5	250	93.1	0.9987	-
	R: TTTGATGTCACGCACGATTT					
*Gapdh*	F: AGGTCGGTGTGAACGGATTTG	NM_001289463.1	123	87.6	0.9999	-
	R: TGTAGACCATGTAGTTGAGGTCA					
*Rn18s*	F: GCAATTATTCCCCATGAACG	NR_003278.3	123	90.3	0.9856	[22]
	R: GGCCTCACTAAACCATCCAA					
*Hmbs*	F: ATGAGGGTGATTCGAGTGGG	NM_001110251.1	134	88.9	0.9864	[25]
	R: TTGTCTCCCGTGGTGGACATA					
*Ppia*	F: CAGGTCCATCTACGGAGAGA	NM_008907.2	146	90.3	0.9990	[30]
	R: CATCCAGCCATTCAGTCTTG					
*B2m*	F: ATACGCCTGCAGAGTTAAGC	NM_009735.3	70	87.9	0.9838	[30]
	R: TCACATGTCTCGATCCCAGT					
*Tbp*	F: CCAATGACTCCTATGACCCCTA	NM_013684.3	104	98.8	0.9998	[26]
	R: CAGCCAAGATTCACGGTAGA					
*Tfrc*	F: GTTTCTGCCAGCCCCTTATTAT	NM_011638.4	152	102.2	0.9994	[25]
	R: GCAAGGAAAGGATATGCAGCA					
*Nono*	F: TGCTCCTGTGCCACCTGGTACTC	NM_023144.2	170	94.5	0.9995	[23]
	R: CCGGAGCTGGACGGTTGAATGC					
*Rpl13a*	F: GGCTGCCGAAGATGGCGGAG	NM_009438.5	131	93.8	0.9995	[23]
	R: GCCTTCACAGCGTACGACCACC					

### Creation of a heatmap for gene expression levels and the evaluation of 10 reference genes using five algorithms

Heatmap images of the expression of the 10 genes included in the analysis of the ND and DI groups were generated using Heatmapper [34]. Z-scores for the expression levels of each gene were calculated, and genes with higher and lower than the average expression levels are marked in red and green, respectively. The Average Linkage clustering and Pearson distance measurement methods were used. The expression stability of each gene was evaluated using the geNorm (https://cellcarta.com/genomic-data-analysis/ [35]), NormFinder (https://www.moma.dk/software/normfinder [36]), BestKeeper (http://www.gene-quantification.de/bestkeeper.html [37]), and RefFinder (http://www.ciidirsinaloa.com.mx/RefFinder-master/ [38]). In the ∆Ct approach, the stability level was manually calculated using the comparative ∆Ct method proposed by Silver *et al.* [39].

### Statistical analysis

For the expression levels of reference genes and the determination of target gene expression levels normalized against the reference genes, the data are presented as the mean ± standard deviation (SD). Comparisons between the reference gene mRNA expression levels in each group on days 5 and 10 (both ND and DI groups) and those on day 0 (control group) were performed using Dunnett’s test. For the semi-quantitative analysis of oil droplet accumulation and *Lep*, *Pparg*, and *Cebpa* expression analyses, comparisons between means were performed using the Bonferroni test. Microsoft Excel add-in statistical software (Bell Curve for Excel version 4.02; Social Survey Research Information, Tokyo, Japan) was used to perform the statistical analyses. Differences were considered statistically significant at *P < 0.05 and **P < 0.01.

## Results

### Verification of adipocyte differentiation after adipogenic induction

**Figure 1.**
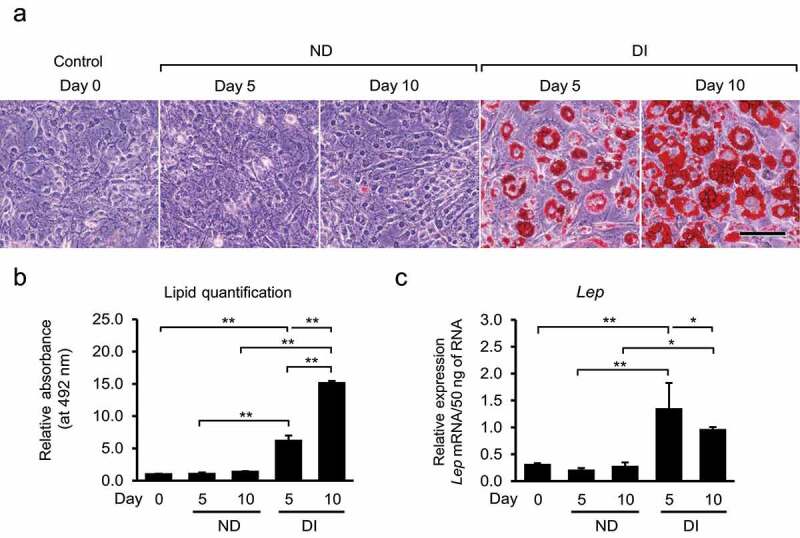
Oil Red O (ORO) staining and quantification of accumulated lipid droplets in non-differentiated (ND) and differentiated (DI) 3T3-L1 cells on days 0, 5, and 10. (a) Accumulation of lipid droplets was observed in the DI groups by staining with ORO, at days 5 and 10 after differentiation induction. Scale bar = 100 µm. (b) Quantification of lipid droplet accumulation in 3T3-L1 cells. The absorbance of the eluted ORO obtained from the stained oil droplets was measured using a microplate reader at 492 nm. *P < 0.05 and **P < 0.01 using Bonferroni test. (c) Relative *Lep* mRNA expression per 50 ng of RNA in all five groups. *Lep* expression was higher in the DI groups than in the ND groups, indicating the stage of mature adipocytes in the DI groups. *P < 0.05 and **P < 0.01 using Bonferroni test. Data are presented as the mean ± standard deviation (SD) of triplicate samples.

In the present study, 3T3-L1 cells from the DI group exhibited morphological changes to adipocyte-like structures, with lipid droplet accumulation observed on days 5 and 10 after adipogenic induction. In contrast, cells in the ND group did not show lipid droplet accumulation, similar to those in the control group (Figure 1(a)). Semi-quantitative measurements showed that the DI group had 6.2-fold higher lipid droplet accumulation than the control group on day 5, and 15.1-fold higher accumulation on day 10 (Figure 1(b)). Additionally, *Lep* mRNA expression was higher in the DI group on days 5 and 10, indicating the presence of mature adipocytes (Figure 1(c)). Induction of differentiation triggers deep phenotypic changes in preadipocytes as they transition into mature adipocytes. In contrast, the cells that were not subjected to differentiation induction maintained their fibrocyte-like phenotypes.

### Different reference gene expression levels in non-differentiating and differentiating 3T3-L1 cells

**Figure 2.**
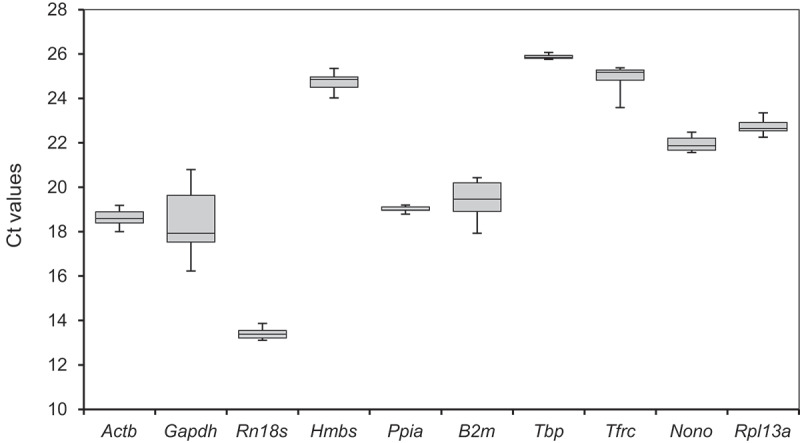
Distribution of cycle threshold (Ct) values of each candidate reference gene across all sample groups. A line within each box indicates the median Ct values, whereas the lower and upper boundaries of each box indicate the first and third quartiles of the data, respectively. Minimum and maximum values are indicated by the lower and upper ends of the whiskers, respectively.

**Figure 3.**
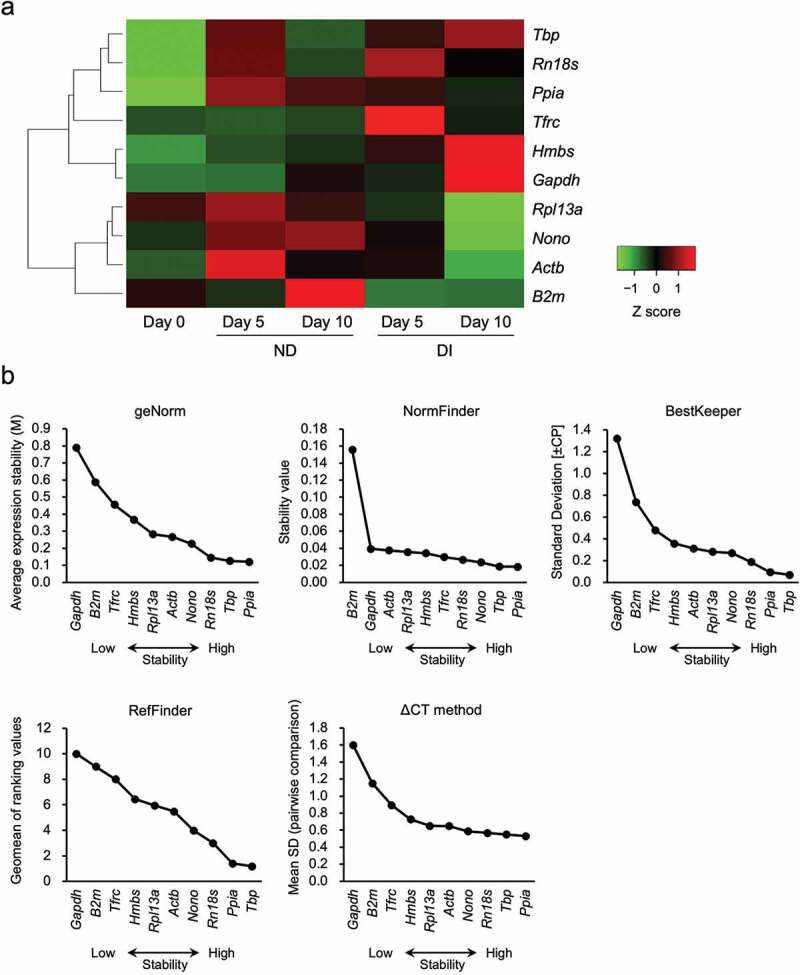
Heatmap of the gene expression levels of 10 reference genes and the gene expression stability levels evaluated using five algorithms. (a) Heatmap image for each gene expression level in the control, non-differentiated (ND), and differentiated (DI) groups. The colours in the heatmap show the Z-scores of gene expression levels in the triplicate samples. Gene expression levels higher than the mean are indicated in red and those lower than the mean are indicated in green. (b) The expression levels of 10 reference genes were analysed using geNorm, BestKeeper, NormFinder, RefFinder, and the ΔCt method in the control, ND, and DI groups. A lower value (right side of the horizontal axis) indicates a reference gene with a more stable expression.

The cycle threshold (Ct) values of the 10 reference genes and their gene expression levels, as calculated from the calibration curves, are provided in Supplementary Tables S1 and S2. The candidate reference genes showed a wide range of Ct values of 10 reference genes across all five sample groups, ranging from 13.11–26.07. *Rn18s* and *Gapdh* exhibited the highest expression levels, with median Ct values of 13.38 and 17.93, respectively. In contrast, *Tbp* showed the lowest expression, with a median Ct value of 25.85 (Figure 2). In addition, *Ppia* and *Tbp* exhibited relatively smaller ranges of Ct values than other candidate genes. We observed fluctuations in the expression levels of the 10 reference genes at different time points (0, 5, and 10 days), regardless of their exposure to differentiation induction, as shown in the Z-score heatmap (Figure 3(a)). Furthermore, these results show for the first time that the expression levels of reference genes vary at different time points in non-differentiated 3T3-L1 cells as well as in differentiation-induced cells.

### Validation of suitable reference genes using five algorithms

The stability of the expression levels of the 10 candidate reference genes was analysed to determine the most suitable reference genes for 3T3-L1 adipocyte studies under both non-differentiating and differentiating conditions. The results of the gene expression stability ranking analysis using the five algorithms are presented in Table 2. geNorm evaluates the reference gene stability (*M*), defined as the average pairwise variation in the expression levels of a certain gene and all candidate genes [35]. A lower *M* value obtained using the geNorm algorithm indicates more stable gene expression. The genes that exhibited the most stable expression in both non-differentiating and differentiating 3T3-L1 cells (days 0, 5, and 10) were *Ppia* and *Tbp*. The expression stabilities of the remaining candidate genes, from highest to lowest, were *Rn18s*, *Nono*, *Actb*, *Rpl13a*, *Hmbs*, *Tfrc*, *B2m*, and *Gapdh* (Figure 3(b), Table 2).

**Table 2.** Gene expression stability ranking by five different algorithms on non-differentiated and differentiation induced 3T3-L1 cells.

		geNorm		NormFinder		BestKeeper		RefFinder		ΔCt method	
	Geometric mean of ranks	*M* value	Rank	Stability value	Rank	Std Dev [± CP]	Rank	Geomean of ranking values	Rank	Mean SD	Rank
*Ppia*	1.320	0.121	1	0.018	1	0.094	2	1.4	2	0.532	1
*Tbp*	1.516	0.126	2	0.019	2	0.069	1	1.2	1	0.549	2
*Rn18s*	3.178	0.145	3	0.027	4	0.187	3	3.0	3	0.567	3
*Nono*	3.776	0.227	4	0.023	3	0.268	4	4.0	4	0.587	4
*Actb*	5.697	0.267	5	0.038	8	0.312	6	5.5	5	0.649	5
*Rpl13a*	5.966	0.283	6	0.036	7	0.281	5	6.0	6	0.650	6
*Hmbs*	6.787	0.368	7	0.034	6	0.356	7	6.4	7	0.727	7
*Tfrc*	7.282	0.456	8	0.030	5	0.479	8	8.0	8	0.895	8
*B2m*	9.192	0.589	9	0.156	10	0.737	9	9.0	9	1.151	9
*Gapdh*	9.791	0.791	10	0.039	9	1.322	10	10.0	10	1.600	10

NormFinder automatically determines the stability values for all candidate reference genes analysed in any number of samples arranged into any number of experimental groups [36]. The NormFinder algorithm-derived stability values of the 10 reference genes shown in Figure 3(b) and Table 2 demonstrate that *B2m* had the least stable gene expression, with a stability value 4-fold higher than that of the second most unstable gene, *Gapdh*. The gene expression stability ranking of the 10 reference genes from NormFinder showed that *Ppia* and *Tbp* had the most stable gene expression (Table 2). Although the expression of the middle-ranked reference genes varied slightly, the genes with the most and least stable expression identified using NormFinder were consistent with the results obtained using geNorm, as mentioned previously.

BestKeeper evaluates the expression stability of reference genes by calculating the SD of Ct values and the coefficient of variation, followed by a pairwise correlation analysis of the BestKeeper index and an analysis of the correlation between the expression levels of each candidate gene [37]. Based on the analysis performed using the other three algorithms and the comparative ΔCt method, RefFinder provides geomean ranking values [38]. As shown in Figure 3(b), the results generated by the BestKeeper and RefFinder programs validate the results of the other algorithms. According to these algorithms, the gene with the most stable expression was *Tbp*, followed by *Ppia*, whereas the genes with the most unstable expression were *Gapdh* and *B2m*. Manual calculations using the comparative Ct method gave results similar to those generated by the other four algorithms, with *Ppia* and *Tbp* as the genes with the most stable expression and *Gapdh* and *B2m* with the least stable expression (Figure 3(b)). Moreover, the ranking of gene expression stabilities obtained using the Ct method was the same as that obtained using the geNorm method (Table 2).

The ranking of the expression stability of the reference genes analysed in this study is shown in Table 2. The geometric means of the ranks calculated from these five algorithms indicated that *Ppia* and *Tbp* had the highest ranks for expression stability, whereas *Gapdh* and *B2m* had the least stable expression. Thus, *Ppia* and *Tbp* could be considered the most suitable reference genes for gene expression studies under both non-differentiating and differentiating conditions. However, *Gapdh* and *B2m* might not be suitable for studying gene expression in 3T3-L1 cells.

### Impact of reference gene selection on the interpretation of the expression levels of target genes

**Figure 4.**
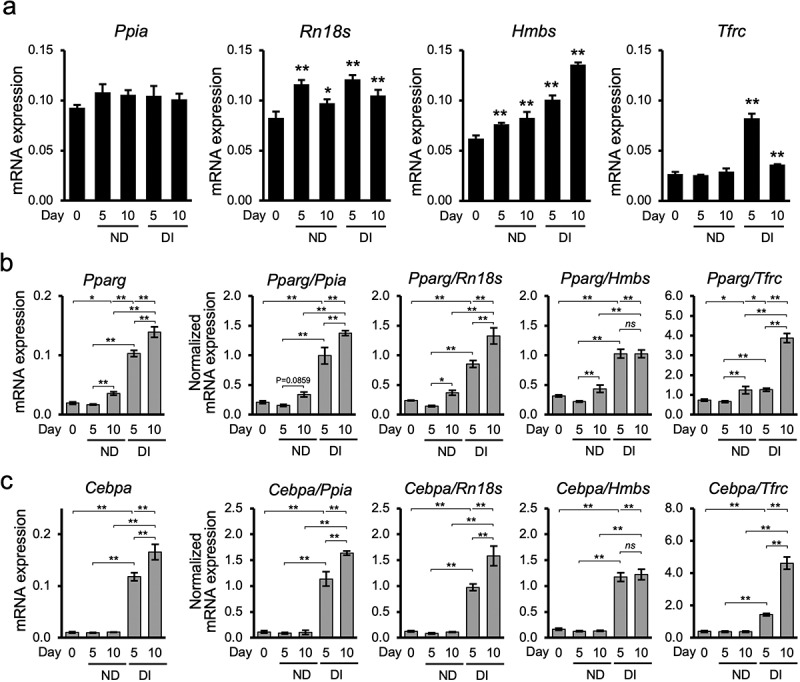
Selection of reference genes affects the expression pattern of the target genes. (a) Expression pattern of representative stable and unstable reference genes in non-differentiating and differentiating 3T3-L1 cells. The non-normalized expression levels of *Ppia*, *Rn18s*, *Hmbs*, and *Tfrc* show different patterns indicating that the gene expression levels differed among the groups. Differences were considered statistically significant at *P < 0.05 and **P < 0.01 compared to the Control group using Dunnett’s test. (b) Comparative gene expression levels of *Pparg* in non-differentiated (ND) and differentiated (DI) groups against the representative gene expression levels. *P < 0.05 and **P < 0.01 using Bonferroni test. (c) *Cebpa* expression levels in non-differentiated (ND) and differentiated (DI) groups normalized to the selected reference genes. *P < 0.05 and **P < 0.01 using Bonferroni test. Data for all graphs are presented as the mean ± standard deviation (SD) of triplicate samples.

In the present study, gene expression levels calculated from the calibration curve were used to prepare graphs. Our results showed that the expression patterns of the reference genes in 3T3-L1 cells might be altered not only in differentiating cells owing to adipogenic induction but also in non-differentiating cells (Figure 4(a)). *Ppia* was the gene with the most stable expression in 3T3-L1 cells. *Rn18s* expression increased on day 5 and decreased slightly on day 10, regardless of adipogenic induction. The expression levels of *Hmbs* increased in a day-dependent manner and accelerated in the DI group. In addition, the expression levels of *Tfrc* were relatively stable in the ND group; however, their expression levels increased with adipogenic induction on day 5 and then slightly decreased on day 10. The expression patterns of the other six reference genes are shown in Supplementary Figure S1. *Tbp*, the gene with the second most stable expression, exhibited a stable expression pattern, in addition to *Ppia*. On day 10, *Nono*, *Actb*, and *Rpl13a* showed decreased expression levels. Despite the significance levels in comparison with the control group, their expression was relatively lower in the DI group than in the ND group. *B2m* expression levels were lower in the ND group on day 5, followed by a significant increase on day 10, whereas its expression level remained consistently lower in the DI group. *Gapdh*, the gene with the least stable expression, showed an increase in gene expression in a day-dependent manner, which was more significant in the DI group.

In the present study, we normalized two adipogenesis-related transcription factors, *Pparg* and *Cebpa* [40], against four selected reference genes, *Ppia*, *Rn18s*, *Hmbs*, and *Tfrc*, which have expression patterns with different stabilities ranging from more to less stable (Figure 4(b,c)). This was performed to demonstrate how the selection of reference genes affected the results of the gene expression analysis. The expression of PPARG and CEBPA cooperatively induces adipogenesis [40]. These genes exhibit similar expression patterns. The expression level of *Pparg* in the DI group was significantly increased on days 5 and 10 after adipogenic induction. Specifically, the raw expression level of *Pparg* was 1.35-fold higher on day 10 than on day 5 after induction. When the expression level of *Pparg* was normalized to that of *Ppia* and *Rn18s*, *Pparg* expression increased by 1.38- and 1.55-fold, respectively. These expression patterns are consistent with the raw expression patterns (Figure 4(b)). However, the magnitude of *Pparg* expression on day 10 in the DI group, when normalized to *Hmbs* expression levels, did not increase, unlike the raw expression profile. Generally, *Pparg* expression levels were markedly increased compared to the raw expression levels when normalized to *Tfrc*, whose expression levels were relatively low. Moreover, *Pparg* expression levels in the DI group were 3.08-fold higher on day 10 than on day 5 after induction. These consistent and inconsistent expression patterns compared to the raw expression pattern were also observed for *Cebpa* (Figure 4(c)). *Pparg* and *Cebpa* expression levels normalized to those of *Tbp*, *Nono*, *Actb*, *Rpl13a*, *B2m*, and *Gapdh* are presented in Supplementary Figure S2. On day 10, the DI group showed a 1.31-fold increase in *Pparg* expression levels normalized to *Tbp* compared to day 5, which was similar to the non-normalized expression level. Additionally, *Pparg* expression levels normalized to *Nono*, *Actb*, and *Rpl13a* in the DI group were 1.81-, 2.03-, and 1.83-fold higher, respectively, on day 10 than on day 5 after adipogenic induction. Normalizing *Pparg* expression against the genes with the least stable expression, *B2m* and *Gapdh*, resulted in distinctive expression patterns compared to non-normalized expression. As *Pparg* and *Cebpa* mRNA levels were similar in 3T3-L1 cells, the correct and incorrect patterns of *Cebpa* normalized against *Tbp*, *Nono*, *Actb*, *Rpl13a*, *B2m*, and *Gapdh* were similar to those of *Pparg* (Supplementary Figure S2).

## Discussion

The strategy for normalizing the expression levels of a gene of interest against appropriate reference genes in RT-qPCR gene expression analysis is based on the assumption that reference genes are expressed at constant levels across various treatments and experimental conditions [41]. However, an ideal reference gene does not exist [18,35,42]. Disparities in gene expression levels were observed at different time points for all housekeeping genes analysed in this study, regardless of adipogenic induction, as shown in the Z-score heatmap (Figure 3(a)). The most common reference genes, such as *Gapdh*, *Actb*, and *Rn18s* are often used to normalize gene expression data in RT-qPCR analysis. Commonly used reference genes, such as *Gapdh* and *Actb*, are acceptable for qualitative or semi-quantitative assays, including conventional RT-PCR [18]. However, several studies have demonstrated that some commonly used reference genes such as *Actb*, *Rn18s*, and *Gapdh* are unstable in different tissues under various experimental conditions [43,44].

In a previous study of 3T3-L1 differentiation, *Rn18s* and *Hmbs* were the most stable reference genes [25]. *Hmbs* and *B2m* are the most stable reference genes for 3T3-L1 adipocyte differentiation under hypoxic and normoxic conditions [22]. Other research groups have recommended normalization against *Atp5b*, *Nono*, and *Hprt* as the most suitable reference genes during the mitotic clonal expansion (MCE) phase of 3T3-L1 differentiation, whereas *Actb*, *Nono, Rpl13a*, and *Ywhaz* have been reported as the best reference genes during the terminal differentiation phase [23]. From our perspective, appropriate reference genes may differ between studies depending on the experimental design. However, the use of commonly used reference genes without validation before gene expression analyses has yielded unreliable results. Therefore, the normalization of gene expression levels against inappropriate reference genes may have a major impact on the conclusions of the study owing to false statistical analysis results and misinterpretation of data [24].

Unlike previous validations, the present study evaluated the expression of 10 candidate reference genes, not only in adipogenic-induced 3T3-L1 cells but also in non-differentiated cells during the culture period. A comparison of gene expression between differentiating and non-differentiating cells, which were maintained for the same culture period, is necessary to determine whether the alteration in gene expression was caused by adipogenic induction or whether it was naturally altered during the culture period (day 0–10). In the present study, all five algorithms showed that *Gapdh* and *B2m* were unsuitable because their expression levels were unstable across the experimental groups. Despite its common utilization as a reference gene in gene expression analysis, *Gapdh* mRNA is stimulated by insulin on 3T3-Ll and 3T3-F442A cell lines [45,46], as well as upregulated during rat brown adipocytes differentiation [47]. This suggests that *Gapdh* must be avoided in in vitro adipogenesis studies because insulin is frequently used in adipocyte culture media [14].

Our study also validated *Ppia* and *Tbp* as the most suitable reference genes for 3T3-L1 adipogenesis studies comparing differentiating and non-differentiating cells. As shown in Figure 4(b,c), we demonstrated the importance of selecting an appropriate reference gene. Normalization against *Ppia* resulted in consistent *Pparg* and *Cebpa* expression levels compared with their non-normalized expression levels (Figure 4(b,c)). Similar results were obtained when expression levels were normalized to those of *Tbp* (Supplementary Figure S2). In addition to the top-ranked reference genes *Ppia* and *Tbp*, we demonstrated that normalization to *Rn18s* was acceptable for determining *Pparg* and *Cebpa* expression levels. Although *Rn18s* expression is not the most stable, the results of this study are consistent with those of a previous study [25]. The difference in *Pparg* and *Cebpa* expression levels between differentiated and non-differentiated cells was extremely significant, and the surge in gene expression levels was also significantly different between the time points. Therefore, normalization to unstable reference genes may not detect any changes in the gene expression patterns. This result emphasized that reference gene suitability may also affect target gene expression patterns. The present study showed that the expression of the unstable reference gene *Hmbs*, increased in a day-dependent manner, regardless of the presence or absence of differentiation induction, whereas Zhang *et al.* [25] revealed consistent expression of *Hmbs* during differentiation. Normalization of *Pparg* and *Cebpa* expression against *Hmbs* showed no difference in expression levels between undifferentiated and differentiated cells, resulting in incorrect expression patterns (Figure 4(b,c)). In the determination of *Pparg* and *Cebpa* expression levels, normalization against *Tfrc* showed extremely high gene expression levels with a similar statistical justification; however, this resulted in incorrect data (Figure 4(b,c)). Moreover, normalization against other unstable reference genes, namely, *Nono*, *Actb*, *Rpl13a*, *B2m*, and *Gapdh*, resulted in inaccurate expression levels (Supplementary Figure S2). Taken together, these data highlight that normalizing gene expression levels to those of an inappropriate reference gene can lead to misinterpretation of data.

Gene expression variability may be affected by various factors, including glucose concentration [48,49], FBS supplementation [50,51], pH [52,53], and temperature [54,55]. In addition, gene expression variability is caused by the cell passage number [56,57], culture duration [58,59], cell cycle synchronization [60], and experimental treatments [61–63]. Among these factors, the physicochemical environment (pH and temperature) is accurately controlled. The biochemical conditions of the cellular environment in this study were maintained using the same cell culture medium throughout the experiments. A constant glucose concentration, a single batch of FBS contained in the culture media, and the implementation of a routine media replacement protocol eliminated the possibility of these factors causing gene expression variability. Cells at the same passage number were used to ensure homogeneity of the physical characteristics of the cells among all experimental groups. However, in this study, we did not synchronize the cell cycle before day 0. Therefore, an unsynchronized cell cycle might affect gene expression, especially in the ND groups. Nonetheless, our study revealed that some reference genes were stably expressed across the different treatments and throughout the experimental period. Gene expression variability was likely caused by treatment with adipogenic induction reagents and the experimental duration, rather than by a lack of synchronization.

We showed that the expression levels of reference genes may be affected over time, even in non-differentiating cells, and that *Ppia* and *Tbp* were stable reference genes throughout 10 days in both undifferentiated and differentiated 3T3-L1 cells. One limitation of this study is that it focused on only 10 reference genes; thus, reference genes other than those analysed in this study may be suitable under a similar experimental design. Notably, the expression of known reference genes was altered in non-differentiating cells throughout the experiment.

## Supplementary Material

Supplemental Material

## Data Availability

All relevant data supporting the key findings of this study are available within the article and its Supplementary Information files, or from the corresponding author upon reasonable request. The data compiled and calculated as part of this study are available from the BioStudies database (https://www.ebi.ac.uk/biostudies) under accession number S-BSST1110.
