# Supplementary material for: Housekeeping gene expression variability in differentiating and non-differentiating 3T3-L1 cells
Source: Adipocyte. 2023 Jul 20;12(1):2235081. doi: 10.1080/21623945.2023.2235081 (PMC10364660; doi:10.1080/21623945.2023.2235081)
Supplement: Supplemental Material [file KADI_A_2235081_SM9144.zip › Revised Supplementary Figures S1 and S2.docx]

# a

0.15

mRNA expression

0.10

0.05

0.00

## Tbp

b

0.20

mRNA expression

0.15

0.10

0.05

0.00

## Nono

c

0.25

mRNA expression

0.20

0.15

0.10

0.05

0.00

## Actb

**

*

*

Day 0

# d

0.25

**

mRNA expression

0.20

0.15

0.10

0.05

### 5 10 5 10

ND DI

*Rpl13a*

Day 0

# e

0.80

**

**

* * **

mRNA expression

0.60

0.40

0.20

### 5 10 5 10

ND DI

*B2m*

Day

# f

0.20

**

**

*

mRNA expression

0.15

0.10

0.05

### 0 5 10 5 10

ND DI

*Gapdh*

0.00

### Day 0 5 10 5 10

ND DI

0.00

### Day 0

5 10

ND

### 5 10

DI

0.00

### Day 0

5 10

ND

### 5 10

DI

# a

2.0

** ** **

**

**

**

**

Normalized mRNA expression

1.5

1.0

0.5

0.0

Day

2.5

** **

**

**

**

Normalized mRNA expression

2.0

1.5

1.0

0.5

0.0

#### Pparg/Tbp

0 5 10 5 10

ND DI

#### Pparg/Rpl13a

2.5

2.0

Normalized mRNA expression

1.5

1.0

0.5

0.0

Day

2.0

** **

**

**

**

Normalized mRNA expression

1.5

1.0

0.5

0.0

#### Pparg/Nono

0 5 10 5 10

** **

**

**

**

ND DI

#### Pparg/B2m

2.5

2.0

Normalized mRNA expression

1.5

1.0

0.5

0.0

Day

10.0

Normalized mRNA expression

8.0

6.0

4.0

2.0

0.0

#### Pparg/Actb

0 5 10 5 10

** **

**

**

**

ND DI

#### Pparg/Gapdh

Day

# b

2.5

** **

**

**

**

Normalized mRNA expression

2.0

1.5

1.0

0.5

0.0

0 5 10 5 10

ND DI

#### Cebpa/Tbp

Day

3.0

** **

**

**

**

Normalized mRNA expression

2.5

2.0

1.5

1.0

0.5

0.0

0 5 10 5 10

ND DI

#### Cebpa/Nono

Day 0 5 10 5 10

ND DI

#### Cebpa/Actb

3.0

** **

**

**

**

Normalized mRNA expression

2.5

2.0

1.5

1.0

0.5

0.0

Day

3.0

** **

**

**

**

Normalized mRNA expression

2.5

2.0

1.5

1.0

0.5

0.0

Day

0 5 10 5 10

ND DI

#### Cebpa/Rpl13a

0 5 10 5 10

ND DI

Day 0 5 10 5 10

ND DI

#### Cebpa/B2m

2.0

** **

**

**

**

Normalized mRNA expression

1.5

1.0

0.5

0.0

Day 0 5 10 5 10

ND DI

Day 0 5 10 5 10

ND DI

#### Cebpa/Gapdh

10.0

Normalized mRNA expression

8.0

6.0

4.0

2.0

0.0

Day 0 5 10 5 10

ND DI
