# Supplementary material for: Housekeeping gene expression variability in differentiating and non-differentiating 3T3-L1 cells
Source: Adipocyte. 2023 Jul 20;12(1):2235081. doi: 10.1080/21623945.2023.2235081 (PMC10364660; doi:10.1080/21623945.2023.2235081)
Supplement: Supplemental Material [file KADI_A_2235081_SM9144.zip › Revised Supplementary Tables S1 and S2.docx]

| **Supplementary Table S1**. The Ct values of non-differentiated and differentiation induced 3T3-L1 cell line | | | | | | | | | | |
| --- | --- | --- | --- | --- | --- | --- | --- | --- | --- | --- |
| Gene | *Actb* | *Gapdh* | *Rn18s* | *Hmbs* | *Ppia* | *B2m* | *Tbp* | *Tfrc* | *Nono* | *Rpl13a* |
| Control, Day 0 (1) | 18.748 | 19.930 | 13.636 | 25.199 | 19.166 | 19.023 | 25.843 | 25.177 | 21.871 | 22.348 |
| Control, Day 0 (2) | 18.982 | 20.800 | 13.868 | 25.354 | 19.105 | 19.033 | 26.072 | 25.380 | 22.017 | 22.653 |
| Control, Day 0 (3) | 18.812 | 19.631 | 13.813 | 25.306 | 19.200 | 18.802 | 25.950 | 25.226 | 22.119 | 22.703 |
| ND Day 5 (1) | 18.004 | 19.884 | 13.209 | 24.990 | 18.968 | 19.656 | 25.879 | 25.313 | 21.664 | 22.623 |
| ND Day 5 (2) | 18.129 | 19.488 | 13.200 | 24.937 | 18.789 | 19.363 | 25.804 | 25.310 | 21.844 | 22.255 |
| ND Day 5 (3) | 18.088 | 19.576 | 13.304 | 24.951 | 18.998 | 19.467 | 25.815 | 25.344 | 21.588 | 22.384 |
| ND Day 10 (1) | 18.741 | 17.647 | 13.560 | 24.949 | 19.026 | 18.169 | 25.958 | 25.253 | 21.705 | 22.510 |
| ND Day 10 (2) | 18.333 | 17.618 | 13.532 | 24.718 | 18.889 | 17.933 | 25.801 | 24.976 | 21.624 | 22.648 |
| ND Day 10 (3) | 18.558 | 17.441 | 13.442 | 24.856 | 18.946 | 18.022 | 25.957 | 25.199 | 21.685 | 22.573 |
| DI Day 5 (1) | 18.520 | 17.932 | 13.195 | 24.553 | 19.113 | 20.396 | 25.763 | 23.642 | 22.304 | 23.140 |
| DI Day 5 (2) | 18.595 | 17.828 | 13.215 | 24.577 | 18.824 | 20.428 | 25.928 | 23.746 | 21.869 | 22.659 |
| DI Day 5 (3) | 18.443 | 19.642 | 13.109 | 24.450 | 18.980 | 20.201 | 25.860 | 23.588 | 21.566 | 22.625 |
| DI Day 10 (1) | 19.092 | 16.472 | 13.319 | 24.026 | 18.970 | 20.194 | 25.805 | 24.810 | 22.480 | 23.294 |
| DI Day 10 (2) | 19.154 | 16.392 | 13.383 | 24.076 | 19.123 | 20.315 | 25.848 | 24.855 | 22.346 | 23.355 |
| DI Day 10 (3) | 19.190 | 16.230 | 13.479 | 24.067 | 18.979 | 20.153 | 25.788 | 24.838 | 22.366 | 23.257 |

| **Supplementary Table S2**. The gene expression levels calculated from calibration curves | | | | | | | | | | |
| --- | --- | --- | --- | --- | --- | --- | --- | --- | --- | --- |
| Gene | *Actb* | *Gapdh* | *Rn18s* | *Hmbs* | *Ppia* | *B2m* | *Tbp* | *Tfrc* | *Nono* | *Rpl13a* |
| Control, Day 0 (1) | 0.140 | 0.011 | 0.090 | 0.066 | 0.092 | 0.361 | 0.123 | 0.028 | 0.144 | 0.202 |
| Control, Day 0 (2) | 0.120 | 0.006 | 0.077 | 0.059 | 0.096 | 0.359 | 0.105 | 0.024 | 0.131 | 0.165 |
| Control, Day 0 (3) | 0.135 | 0.013 | 0.080 | 0.061 | 0.090 | 0.416 | 0.114 | 0.027 | 0.122 | 0.160 |
| ND Day 5 (1) | 0.229 | 0.011 | 0.118 | 0.075 | 0.105 | 0.242 | 0.120 | 0.026 | 0.165 | 0.168 |
| ND Day 5 (2) | 0.211 | 0.014 | 0.119 | 0.077 | 0.117 | 0.292 | 0.126 | 0.026 | 0.147 | 0.215 |
| ND Day 5 (3) | 0.217 | 0.014 | 0.111 | 0.077 | 0.103 | 0.273 | 0.125 | 0.025 | 0.174 | 0.197 |
| ND Day 10 (1) | 0.141 | 0.046 | 0.094 | 0.077 | 0.101 | 0.620 | 0.113 | 0.027 | 0.161 | 0.181 |
| ND Day 10 (2) | 0.185 | 0.047 | 0.096 | 0.089 | 0.110 | 0.719 | 0.126 | 0.032 | 0.170 | 0.166 |
| ND Day 10 (3) | 0.159 | 0.052 | 0.102 | 0.082 | 0.106 | 0.680 | 0.113 | 0.028 | 0.163 | 0.174 |
| DI Day 5 (1) | 0.163 | 0.038 | 0.119 | 0.099 | 0.095 | 0.152 | 0.130 | 0.083 | 0.108 | 0.120 |
| DI Day 5 (2) | 0.155 | 0.041 | 0.118 | 0.097 | 0.115 | 0.149 | 0.116 | 0.077 | 0.144 | 0.164 |
| DI Day 5 (3) | 0.172 | 0.013 | 0.126 | 0.106 | 0.104 | 0.172 | 0.121 | 0.086 | 0.176 | 0.168 |
| DI Day 10 (1) | 0.112 | 0.096 | 0.110 | 0.138 | 0.105 | 0.173 | 0.126 | 0.037 | 0.096 | 0.108 |
| DI Day 10 (2) | 0.107 | 0.101 | 0.106 | 0.134 | 0.095 | 0.160 | 0.122 | 0.035 | 0.105 | 0.104 |
| DI Day 10 (3) | 0.105 | 0.112 | 0.099 | 0.135 | 0.104 | 0.177 | 0.127 | 0.036 | 0.104 | 0.111 |
